# Cabozantinib for the Treatment of Metastatic Medullary Thyroid Carcinoma

**DOI:** 10.6004/jadpro.2014.5.1.10

**Published:** 2014-01-01

**Authors:** Nancy M. Nix, Kirk Braun

**Affiliations:** From St. Joseph’s/Candler Health System, South Carolina Cancer Specialists, Hilton Head, South Carolina; University of Georgia College of Pharmacy, Athens, Georgia

Thyroid cancer is increasing at a faster rate than any other cancer type in the United States, with a total of 60,220 new cases expected in 2013 (American Cancer Society, 2013). It is the fifth most commonly diagnosed cancer in women, but it is much less prevalent in men. Thyroid cancer is generally associated with a high cure rate, yet medullary thyroid carcinoma (MTC) in particular is more aggressive and tends to metastasize.

Medullary thyroid carcinoma constitutes approximately 5% of all thyroid cancers. It arises from the parafollicular cells, also known as the C cells, which are responsible for the production of the hormone calcitonin (Wells et al., 2012). Therefore, calcitonin is a significant tumor marker for perioperative and postoperative prognoses (Ismailov & Piulatova, 2004). The RET (rearranged during transfection) proto-oncogene test can detect a predisposition to the familial malignancy, but 75% of cases are sporadic and may have no genetic component (Wells et al., 2012). The overall survival rate for patients with MTC is 83% at 10 years and 50% at 20 years (Ball, 2007). As is true of all malignancies, early diagnosis improves prognosis.

Thyroidectomy is the primary treatment and the only cure for MTC (National Cancer Institute [NCI], 2013). Although surgery offers a cure for localized disease, distant tumor recurrence is probable in 4% of patients following complete surgical resection (Howlader et al., 2013). Distant disease decreases 5-year overall survival to 55%, so there remains a need to combat MTC beyond surgical measures. When metastatic disease is present, radiation therapy can be used (NCI, 2013). Palliative chemotherapy is available for unresectable or metastatic MTC in patients with either structurally progressive or symptomatic disease.

In April 2011, vandetanib (Caprelsa) was the first agent approved by the US Food and Drug Administration (FDA) for MTC (Wells et al., 2012). The tyrosine kinase inhibitors (TKIs) sorafenib (Nexavar), sunitinib (Sutent), and pazopanib (Votrient) have been used off-label for this disease as well. However, these medications generally confer a clinical benefit that lasts only weeks to months before the onset of tumor progression (Wilhelm et al., 2004; Yakes et al., 2011). Patients who fail to respond to TKIs may receive IV chemotherapy such as CVD (cyclophosphamide, vincristine, and dacarbazine), but response rates are less than 20% (Niafar, Dabiri, Bozorgi, Niafar, & Gholami, 2011). There is currently no regimen that is considered standard of care for unresectable MTC (NCI, 2013).

In November 2012, cabozantinib (Cometriq), an orally bioavailable inhibitor of multiple tyrosine kinase receptors, received FDA approval for the treatment of progressive metastatic MTC.

## Pharmacology and Dosing

Cabozantinib is an oral inhibitor of multiple tyrosine kinases including RET kinase, epidermal growth factor, hepatocyte growth factor, MET, and vascular endothelial growth factor 2 (VEGF2) receptors (Yakes et al., 2011). These pathways are involved in normal cellular reproduction and angiogenesis, but abnormal functioning can lead to cancer proliferation. Inhibition of protein kinases prevents the phosphorylation of the receptors, which is necessary for the proliferation of cancer cells. This induces apoptosis of cancer cells and suppresses tumor growth, metastasis, and angiogenesis (Niafar et al., 2011). Resistance commonly occurs with TKIs, making a therapeutic option that targets multiple signaling pathways desirable (Yakes et al., 2011). Furthermore, targeting the VEGF receptor alone could promote tumor growth due to compensatory upregulation of MET. By targeting VEGF and MET, cabozantinib blocks the MET-driven resistance to agents that inhibit either target independently (Yakes et al., 2011; Kurzrock et al., 2011).

The recommended starting dose for cabozantinib is 140 mg taken orally once daily, 1 hour before a meal or 2 hours after a meal. Cabozantinib is available in 20- or 80-mg oral capsule formulations dosed at 60, 100, or 140 mg daily, depending upon toxicity. Indications for dose adjustments are summarized in the Table. It is cleared hepatically, but there are no recommendations for dose adjustment in the setting of mild hepatic impairment. However, cabozantinib is not indicated in the setting of moderate-to-severe liver impairment, where bilirubin levels are greater than 1.5 times the upper limit of normal. Few data exist to support dose adjustments in patients with mild-to-moderate renal impairment, and there are no data to support its use in patients with a creatinine clearance below 30 mL/min (Exelixis, 2012).

**Table 1:**
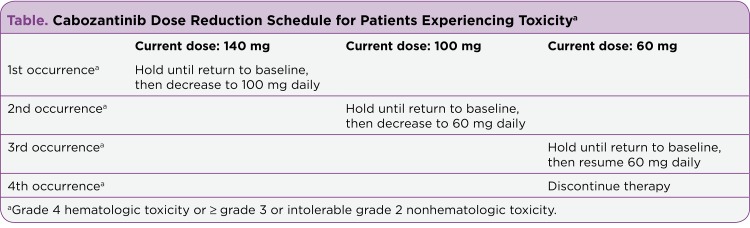
Table 1. Cabozantinib Dose Reduction Schedule for Patients Experiencing Toxicit

Chronic use of strong CYP3A4 inhibitors such as ketoconazole should be avoided with cabozantinib. If these medications are necessary, a 40-mg reduction in the dose of cabozantinib should be implemented. Chronic use of strong CYP3A4 inducers such as phenytoin should also be avoided, but if their use is necessary, the cabozantinib dose should be increased by 40 mg (Exelixis, 2012).

## Clinical Trials

A phase I/II dose escalation study was conducted in 85 patients with MTC, with the primary endpoints being safety, pharmacokinetics, and maximum tolerated dose. To determine the optimal dose and schedule for the phase III study, patients were assigned to 1 of 13 cabozantinib dose levels and 2 treatment schedules. Of these patients, 29% had a confirmed partial response, and 41% had stable disease for at least 6 months.

The most commonly reported adverse events were palmar-plantar erythrodysesthesia (PPE); mucositis; and aspartate transaminase (AST), alanine transaminase (ALT), and lipase elevations. The results of this study indicated that cabozantinib had an acceptable safety profile with good activity against MTC (Kurzrock et al., 2011).

The approval of cabozantinib was based on a phase III, randomized, double-blind, placebo-controlled trial that assessed the safety and efficacy of cabozantinib in 330 patients. Patients with metastatic MTC were randomized to receive either cabozantinib 140 mg or placebo orally once daily. The primary outcome of progression-free survival (PFS) and secondary outcomes of objective response and duration of response were assessed. The majority of the subjects were Caucasian men, with a median age of 55 years.

Prolongation of PFS was observed, with a median PFS of 11.2 vs 4.0 months with placebo (hazard ratio [HR], 0.28; 95% confidence interval [CI] = 0.19–0.40; *p* < .0001). One-year PFS with cabozantinib was 47.3%, vs. 7.2% with placebo. The overall response rate was 27%. The median duration of response was observed to be 14.7 months. However, there was no statistical difference in overall survival (HR, 0.98; 95% CI = 0.63–1.52). Diarrhea, PPE, and nausea were the most common causes of dose reduction, experienced by approximately 80% of patients on cabozantinib (Schöffski et al., 2012).

## Adverse Events

The most common grade 1/2 adverse drug events, occurring in > 10% of patients, included diarrhea, constipation, nausea, stomatitis, PPE, decreased weight, decreased appetite, fatigue, oral pain, hair color changes (hypopigmentation/graying), dysgeusia, hypertension, and abdominal pain. Changes in laboratory values included increased AST and ALT levels, increased alkaline phosphatase, hypocalcemia, thrombocytopenia, neutropenia, thrombocytopenia, hypophosphatemia, and hyperbilirubinemia. Grade 3/4 adverse events necessitating treatment discontinuation occurred in less than 16% of patients. These events included hypertensive crisis, nephrotic syndrome, reversible posterior leukoencephalopathy syndrome, hemorrhage, and visceral perforation or fistula. The most common dose-limiting toxicities were grade 3 PPE and grade 2 mucositis (Exelixis, 2012). As mentioned previously, dose adjustments for toxicity are addressed in the Table.

## Role in Therapy

According to the National Comprehensive Cancer Network (NCCN) treatment guidelines, the current recommendations for MTC are surgical resection with or without radiation therapy, radiation therapy alone, or vandetanib for recurrent and/or unresectable metastatic medullary carcinoma (NCCN, 2013). Cabozantinib, recently added as a category 1, first-line treatment option, is now approved by the FDA for treatment of metastatic MTC based on the phase III trial results. There is currently no recommendation to combine this medication with other therapeutic options or any clear data concerning sequencing. It should be taken orally without food until disease progression or unacceptable toxicities occur (Exelixis, 2012).

## Implications for Advanced Practitioners

Currently, there are limited chemotherapy options for metastatic MTC. Cabozantinib demonstrates that multimodal targeting of both MET and VEGF2 results in a viable treatment option to prevent both metastasis and angiogenesis. This mechanism of action prevents the resistance that could occur with medications that target the VEGF pathway alone and could provide a benefit over current chemotherapy options. This could pave the way for future medications that target these pathways. Cabozantinib will not replace surgery as the gold standard treatment for MTC, but it provides an option in the treatment of MTC when surgery is not feasible. However, patients must take multiple capsules a day, creating a significant pill burden that may contribute to noncompliance. The monthly estimated cost of almost $12,000 may also be a potential limitation of this therapy given the marginal clinical benefit.

## Summary

Cabozantinib has shown efficacy in MTC based on improvement in PFS time compared with placebo in a phase III trial. This medication also has an acceptable side-effect profile for a patient seeking further therapy and a favorable tumor response rate at the recommended dosage of 140 mg. This multitargeted oral TKI provides an important new treatment option for metastatic MTC and sets the stage for further research into therapies that target both MET and VEGF.
